# Study of a Frugal Design Picosecond Laser System for Laser‐Assisted Corneal Surgery

**DOI:** 10.1002/jbio.70356

**Published:** 2026-09-28

**Authors:** Maxime Quirke, Anaïs Langlois, Théo Guilberteau, Pierre Balage, Thomas Hemery, Valentine Saunier, Florent Deloison, Inka Manek‐Hönninger, David Touboul, John Lopez

**Affiliations:** ^1^ University of Bordeaux‐CNRS‐CEA, CELIA UMR Talence France; ^2^ Department of Ophthalmology Rouen University Hospital Rouen France; ^3^ University of Paris‐Est Créteil Créteil Cedex France; ^4^ Helix Surgical Pessac France; ^5^ Polytech Lyon Villeurbanne France; ^6^ ALPhANOV Talence France; ^7^ Department of Ophthalmology, Anterior Segment Unit Bordeaux University Hospital Bordeaux France

**Keywords:** cavitation bubbles, corneal surgery, femtosecond laser, laser‐assisted surgery, picosecond laser

## Abstract

Femtosecond lasers have long been the gold standard for corneal laser‐assisted surgery, but their high cost limits widespread use. This study challenges their supremacy by investigating a cost‐effective alternative: a frugal all‐fiber picosecond laser source. We first conduct a pump‐probe experiment to characterize cavitation bubbles in water based on the laser pulse's temporal profile. Picosecond pulses concentrate energy in a confined volume near the focal point, allowing for more precise material interaction than femtosecond pulses. A distortion in the temporal profile, often seen as a flaw, paradoxically further reduces cavitation bubble size. These findings are then applied to ex vivo porcine cornea cutting tests. Results show that 1.5 ps pulses with a distorted profile create sharp, consistent cuts. This study reveals that a cost‐effective picosecond laser can rival femtosecond lasers in precision, paving the way for more accessible corneal laser‐assisted surgery.

## Graphical abstract

Stromal surface after extraction of the lamella cut with the studied frugal picosecond laser source.

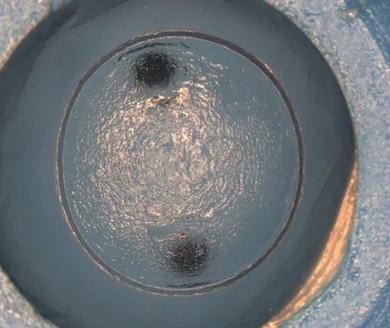

## 1 Introduction

Femtosecond laser technology has become the gold standard in corneal surgery due to its unparalleled precision in tissue ablation. By employing ultrashort pulse durations, femtosecond lasers achieve extremely high peak intensities, which enable accurate disruption of corneal tissue with minimal thermal damage [1, 2, 3, 4]. This effect is attributed to nonlinear multiphoton absorption processes that occur when the laser's pulse duration is on the order of femtoseconds (10^−15^ s). Compared to traditional manual techniques, femtosecond laser‐assisted surgery offers several advantages: it provides better geometric accuracy and superior surface quality of the cut edges [5, 6]. In femtosecond laser‐assisted keratoplasties (KALF), this enhanced precision results in a larger contact area between the graft and the recipient cornea, leading to improved adhesion, enhanced biomechanical stability, and overall better surgical outcomes [7]. The versatility of KALF allows for various edge profiles, including mushroom, top hat, or zigzag configurations, with the latter achievable only through laser assistance. These benefits contribute to superior wound healing, increased resistance to intraocular pressure, faster patient recovery, and reduced rejection rates [8]. Moreover, KALF facilitates quicker suture removal and reduces the risk of postoperative infections due to its minimally invasive nature [9, 10]. Despite these advantages, the high acquisition and maintenance costs of femtosecond laser systems present a significant barrier to broader clinical adoption, particularly in emerging and developing countries.

The introduction of chirped pulse amplification (CPA) by Strickland and Mourou in 1985 was a transformative advancement, leading to the development of femtosecond laser systems capable of cutting a variety of materials and earning them the Physics Nobel Prize in 2018 [11]. However, the field of laser‐assisted corneal surgery has seen limited exploration of picosecond (10^−12^ s) lasers, even though these lasers were highlighted by Stern in 1989 as potentially superior to nanosecond (10^−9^ s) lasers for corneal cutting [12]. Importantly, early research on corneal cutting with ultrashort‐pulse lasers occurred at a time when technologies could not yet explore pulse durations between 500 fs and 20 ps. Between 1989 and 2003, comparisons focused primarily on lasers producing pulses of a few tens of picoseconds and those emitting pulses in the range of hundreds of femtoseconds [13, 14, 15, 16]. The first trials of LASIK flap cutting in humans, published by Krueger et al. in 1998, marked a pivotal moment in corneal surgery, validating the use of ultrashort‐pulse lasers (40 ps) for precise corneal cuts via photodisruption [17, 18]. Picosecond lasers require higher pulse energies compared to femtosecond lasers to achieve photodisruption, which historically led to significant collateral tissue effects. Recent advancements in understanding of laser‐matter interaction, such as those demonstrated by Burakov et al. in fused silica, have shown that picosecond lasers with pulse durations around 2 ps can concentrate energy more densely at the focal point compared to 120 fs, thereby minimizing undesirable effects and potentially improving cutting precision and quality [19].

This study seeks to overcome the primary limitation of current femtosecond laser systems—namely, their high cost—by exploring the potential of a frugal and cost‐effective all‐fiber picosecond laser source developed as a more affordable alternative. We begin by examining cavitation dynamics through pump‐probe experiments conducted in water, chosen for its optical properties analogous to those of the corneal stroma and to mitigate challenges associated with ex vivo corneal tissues. Building on these findings, we will evaluate the performance of the picosecond laser in ex vivo porcine corneas, focusing on key metrics such as cutting precision, edge sharpness, and ablation consistency. By bridging the gap between fundamental laser physics and practical clinical applications, our research seeks to demonstrate how innovations in laser pulse duration and characteristics can lead to more accessible and cost‐effective solutions in corneal surgery.

## 2 Results and Discussion

To explore the capabilities of the frugal picosecond laser source, we first conducted pump‐probe experiments to characterize the cavitation bubbles it generated. We then performed similar analyses with an industrial laser source for comparison. Subsequently, we applied these insights to evaluate the performance of the frugal picosecond laser in corneal cutting tests. All experimental details are given in Section 3.

### 2.1 Pump‐Probe Experiments With the Frugal Picosecond Source

#### 2.1.1 Defining the “Zero Delay” and Initial Observations

Our experiments began by pinpointing the “zero delay” (*t*_0_), defined as the moment when we observe the beginning of a change in the interaction zone. This delay corresponds to a specific position (*x*_0_) on the probe delay line's carriage. With a precision of ±264 fs, we observed the initial modifications, followed by the expansion of the cavitation bubble in the first nanoseconds after its formation for increasing delays in steps of 0.5 ns (Figure 1). By extending the optical path of the probe beyond *x*_0_, we captured the evolution of this process over time, setting the stage for our subsequent measurements.

**FIGURE 1:**
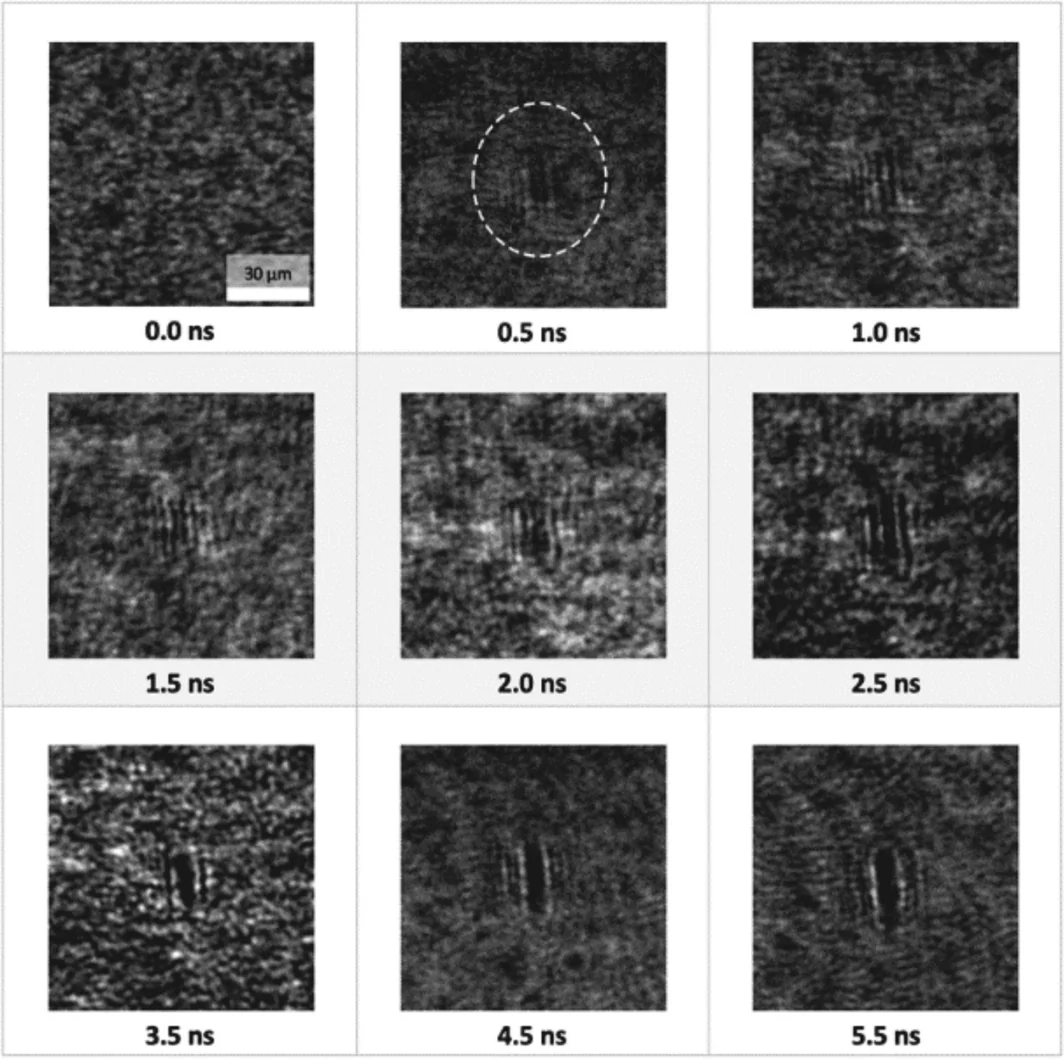
Appearance (white dashed circle) and subsequent expansion of the cavitation bubble over time. Laser beam incident from above.

As shown in Figure 1, bubbles observed in water have an oblong‐like shape which expand over time. This oblong shape is slightly different from the teardrop shape of bubbles observed in water after picosecond and nanosecond laser irradiation [20], or from the teardrop shape of bulk modifications observed in fused silica after femtosecond laser irradiation [21]. This teardrop shape, that corresponds to the energy deposition location within the bandgap material, results from nonlinear propagation effects upstream the focus due to high laser intensity [19, 22, 23]. The teardrop shape corresponds to the early stage of the laser‐water interaction (below 100 ns). It is shown that the bubble shape will gradually become more spherical over time [20, 24].

#### 2.1.2 Relaxation Time Measurement

After defining the “zero delay,” our focus shifted to measuring the relaxation time, defined as the delay between the arrival of the pump pulse at the focal point (i.e., the energy deposition) and the first observable relaxation phenomena (i.e., bubble formation). Indeed, despite the equivalent lengths of the pump and probe optical paths, the appearance of the microbubble occurs after a certain delay after the energy deposition of the pump beam. This delay is measured using an oscilloscope (Waverunner 9404–4 GHz, Teledyne Lecroy) and two identical photodiodes (UPD‐35‐IR2‐P, Alphalas) placed upstream of the focal point and at equivalent distances from the target on the pump and probe lines, respectively, with the probe carriage positioned at *x*_0_. These two photodiodes were connected to the oscilloscope via identical SMA cables to minimize measurement inaccuracies (travel time of electricity in the cables, bandwidth). The relaxation time thus measured is 1.9 ± 1 ns.

#### 2.1.3 Temporal Profile Influence on Cavitation Bubbles

Next, we turned our attention to the temporal profile of the pulses, which significantly influenced the dimensions of the cavitation bubble. Varying the pulse duration from the frugal source also leads to a variation in the contrast of the temporal pulse profile (variation in the *P/A ratio* as defined in Section 3). Therefore, it was impossible to study the effect of these two parameters independently.

We explored pulse durations between 840 and 2060 fs and noted that the median bubble height was smallest for a 1200 fs pulse, with a *P/A ratio* of 19%, at all considered pulse energies (1, 1.5, and 2 μJ on target). We considered the height of the cavitation bubble, rather than its width, based on observations that the bubble width remained relatively constant (ranging between 6 and 12 μm) across all tested parameters, whereas the height exhibited more pronounced and informative variations. These results are presented in Figure 2 in the form of box plots. The box represents the interquartile range (containing 50% of the measured values), the line within the box indicates the median, and the lines extending above and below the box are drawn to the maximum and minimum values, respectively.

**FIGURE 2:**
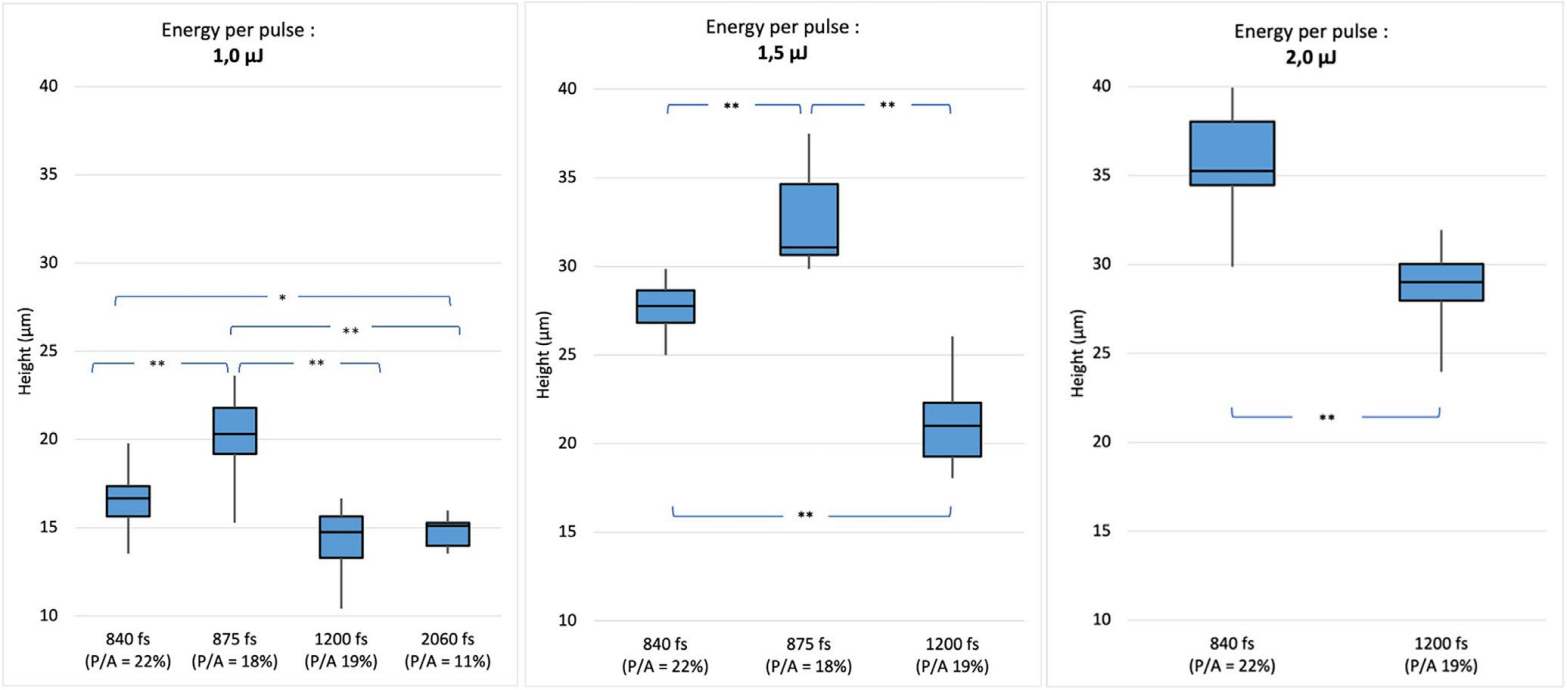
Box plots of bubble heights as a function of pulse duration for each pulse energy value with the frugal source. Statistically significant differences are indicated by the symbols * (*p* < 0.05) and ** (*p* < 0.01).

We observed a statistically significant difference (with an *α* risk of 0.01) between each temporal profile at energies of 1.5 and 2 μJ per pulse, thus supporting the effect of the temporal profile on the size of the cavitation bubble.

Interestingly, these results also show that the variation in cavitation bubble height with pulse duration does not follow a monotonic trend when using the frugal source. This nonlinear behavior contrasts with the observations made using the industrial source, which has a nearly Gaussian temporal profile (i.e., *P/A ratio* < 5%), where the bubble height consistently increased with shorter pulse durations.

From Figure 2, it is possible to determine the mean, the minimum and the maximum bubble dimensions in terms of bubble width $W_{b}$ and bubble height $H_{b}$ (Table 1). From Equation (2) given in Section 3, is it possible to estimate the bubble volume $V_{\text{bubble}}$ (unit is picolitre pL) and the theoretical energy $E_{\text{bubble}}^{\mathrm{vap}}$ (μJ) required to vaporize such a volume of water at ambient temperature and ambient pressure (1 atm). The results are detailed in Table 1. The theoretical energy $E_{\text{bubble}}^{\mathrm{vap}}$ is 1.0 μJ for the smallest bubbles, 3.6 μJ for the mean bubbles, and 9.1 μJ for the largest bubbles. The theoretical energy values are close to pulse energies used in our experiment (1.0, 1.5, and 2.0 μJ). All bubble dimensions given in Figure 2 are measured 4.0 ns after the onset of bubble formation, but as shown in Figure 1, bubbles expand and their dimensions increase over time. This phenomenon results from the gas expansion after laser‐induced cavitation in water. This point may explain the slight difference between the theoretical energy and the experimentally applied pulse energy. It may also explain why the bubble has an oblong‐like shape instead of the theoretical teardrop shape.

**TABLE 1:** Bubble dimensions (in μm), bubble volume (in pL), and corresponding theoretical energy required to evaporate this bubble volume (in μJ).

	Bubble dimensions		
	Mean	Min	Max
$W_{b}\left(\mathrm{\mu m}\right)$	9	6	12
$H_{b}\left(\mathrm{\mu m}\right)$	25	15	35
$V_{\text{bubble}}\left(\mathrm{pL}\right)$	1.4	0.4	3.5
$E_{\text{bubble}}^{\mathrm{vap}}\left(\mathrm{\mu J}\right)$	3.6	1.0	9.1

These bubble dimensions are quite smaller compared to those measured by Vogel et al. (width = 100 μm and height = 500 μm) with longer pulse durations (30 ps and 6 ns) and at a much higher pulse energy (10 mJ) [20].

#### 2.1.4 Impact of Pulse Energy on Bubble Height

To better understand the role of pulse energy, we varied it across a range of 0.9–2.0 μJ for each temporal profile. The results aligned with our expectations: bubble size increased proportionally with pulse energy. For a pulse duration set at 840 fs, bubble height is about 17 μm at 1.5 μJ/pulse, 28 μm at 2.0 μJ/pulse, and 35 μm at 2.5 μJ/pulse. The same trend is observed for longer pulse duration. Indeed, for a pulse duration set at 1200 fs, bubble height is about 15 μm at 1.5 μJ/pulse, 21 μm at 2.0 μJ/pulse, and 29 μm at 2.5 μJ/pulse. These observations corroborated our earlier findings, affirming that the 1.2 ps pulse duration with a 19% *P/A ratio* consistently produced the smallest average bubble size. This step allowed us to establish a broader understanding of how both energy and temporal characteristics shape cavitation characteristics.

#### 2.1.5 The Role of Focusing Depth

We further investigated whether the focusing depth beneath the water surface influenced the bubble dimensions. No significant impact on bubble height or width was found with a focusing depth ranging from 100 to 1000 μm. This consistency across varying depths provided confidence in the robustness of our findings, allowing us to focus on other parameters in subsequent studies.

### 2.2 Pump‐Probe Experiments With the Industrial Source

#### 2.2.1 Modification Threshold

Given that cavitation in water is driven by nonlinear interactions with laser pulses, it was essential to determine the modification threshold—the minimum energy required to visibly alter the material on a nanometer scale. This threshold represents the point where cavitation effects become detectable, offering crucial insight into the interaction dynamics between the laser and the water. We decided to use the industrial laser for this experiment since it offers a wider pulse duration range with a constant contrast of the temporal pulse profile. To identify this threshold, we conducted experiments with pulse durations ranging from 0.5 to 5 ps, while keeping the *P/A ratio* below 5% for all pulse conditions. The results showed a clear linear relationship between the pulse duration and the modification threshold. Specifically, as the pulse duration increased, so did the energy required to induce a visible change. These findings are summarized in Figure 3. Uncertainty on energy measurements is about ±5%.

**FIGURE 3:**
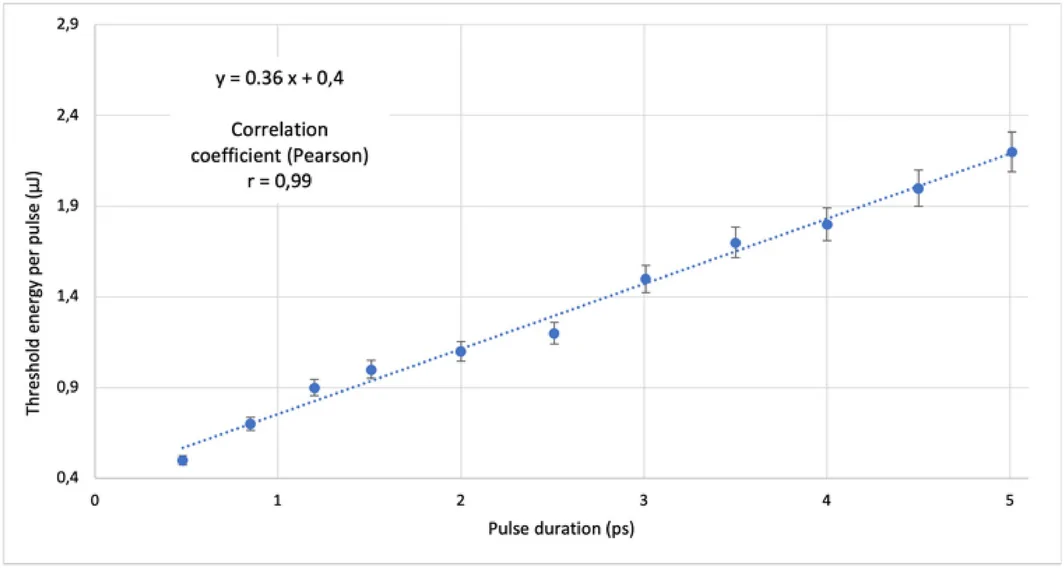
Threshold energy per pulse for micro bubble appearance at various pulse durations.

The linear trend observed suggests that shorter pulse durations are more efficient at inducing cavitation at lower energies, likely due to more concentrated energy deposition over a shorter time scale. From the slope, we can derive a coefficient of 0.36 μJ per ps. Conversely, the longer pulses distribute their energy over a more extended period, requiring greater total energy to reach the same level of material modification. Understanding this relationship between pulse duration and modification threshold is critical for optimizing laser‐based cavitation applications, where precise control over energy deposition is paramount.

#### 2.2.2 Combined Effects of Pulse Energy and Duration

Our next step was to systematically vary pulse energy and duration to map their combined influence on bubble size. For all pulse durations (0.5–5 ps), bubble height increased with pulse energy, showing a near‐linear correlation (Pearson correlation coefficient, *r* > 0.92).

Moreover, when increasing pulse duration at constant energy, we observed a near‐linear decrease in bubble size (*r* ≤ −0.94). Interestingly, across investigated span of parameters, bubble height varies by a factor of 8 whereas bubble width changes by a factor of 2 (6–12 μm), prompting us to focus on bubble height moving forward. These results were consolidated into a three‐dimensional graph (Figure 4), which revealed how pulse energy and duration collectively shape cavitation characteristics.

**FIGURE 4:**
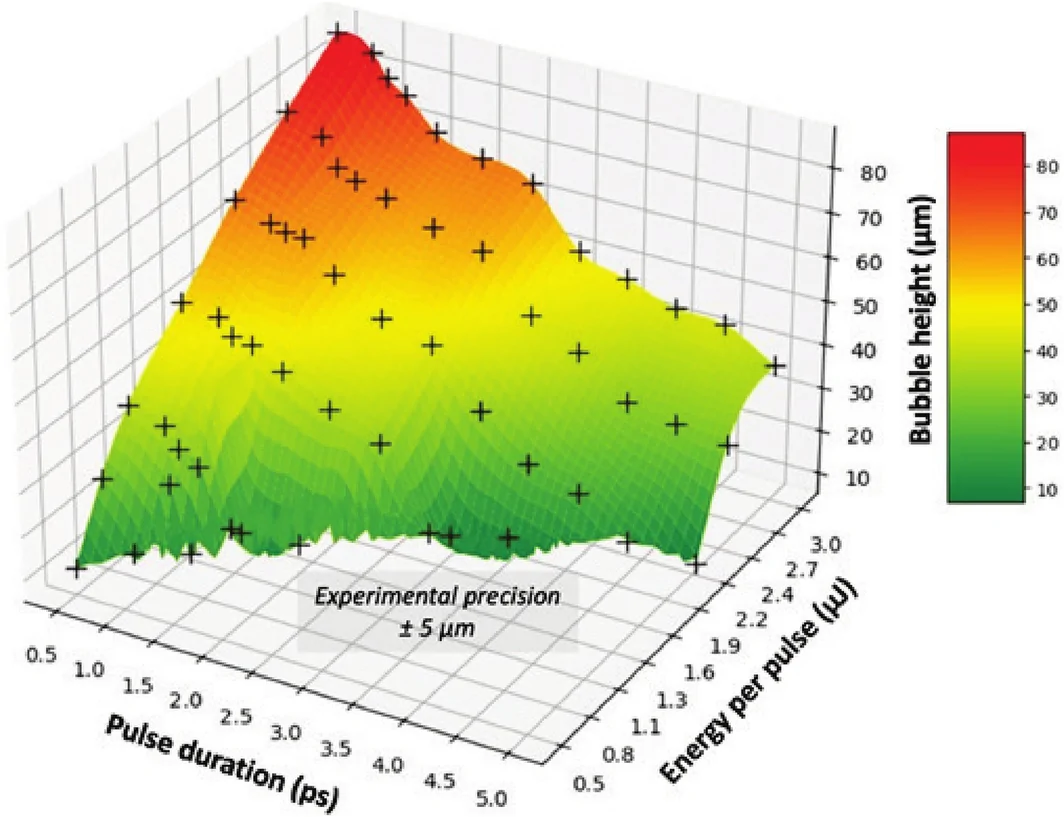
Cavitation bubble height as a function of pulse duration and energy per pulse with the industrial source. Colored surface generated by interpolation from experimental data (+).

From these data, we derived a polynomial regression formula that predicts bubble height based on pulse energy and duration under our experimental conditions:

(1) $$z\;\left(t,E\right)=-1.35-24.45t+56.52E+0.71t^{2}-8.38E^{2}+3.25\mathrm{tE}$$

where *z* is the height of the bubble (in μm), *t* is the pulse duration (in ps), and *E* is the pulse energy (in μJ).

This model closely aligned with our experimental measurements, showing a median absolute error of 2.9 μm and a mean absolute error of 4.3 μm—both below our estimated measurement error (±5 μm). This formula offers a reliable tool for predicting bubble size in future studies.

### 2.3 Exploratory Corneal Ablation

Finally, we turned to the application of these insights in corneal ablation. Our goal was to evaluate both the ease of extraction of the laser cut corneal lamella and the quality of the cuts achieved under different pulse durations and step sizes with the frugal source. By systematically examining these factors, we aimed to optimize the laser parameters for superior surgical outcomes in corneal grafting.

It should be noted that the pulse durations used in this section differ from those in Section 2.1. This variation was motivated by our intention to explore the cutting capabilities across a range of temporal profiles within the picosecond regime, providing a broader understanding of how different pulse durations affect cutting efficiency and quality. Furthermore, the pulse energy used in this section is higher than the range investigated in Figure 4. This is due to the limitations of the experimental setup, where the pump‐probe configuration delivered lower energy on target, making it challenging to achieve higher energies like 5 μJ using the frugal source. However, the pulse energy levels selected in this section were still appropriate for evaluating the cutting capabilities and ensuring system stability.

In terms of cutting quality, optimal results were achieved with a 1.5 ps pulse duration and a step size of 2.5 μm (Figure 5). At 1.5 ps, increasing the step size to 5 μm revealed some rare irregularities visible under the optical microscope, while a step size of 10 μm showed irregularities over nearly 20% of the surface, including some central ones. At 2.8 ps, cutting quality remains very high with a 2.5 μm step size, degrades with a 5 μm step size showing a few peripheral irregularities on about 10% of the surface, and is poor with a 10 μm step size. These results are summarized in Tables 2 and 3 in terms of ease of extraction and cutting quality, respectively.

**FIGURE 5:**
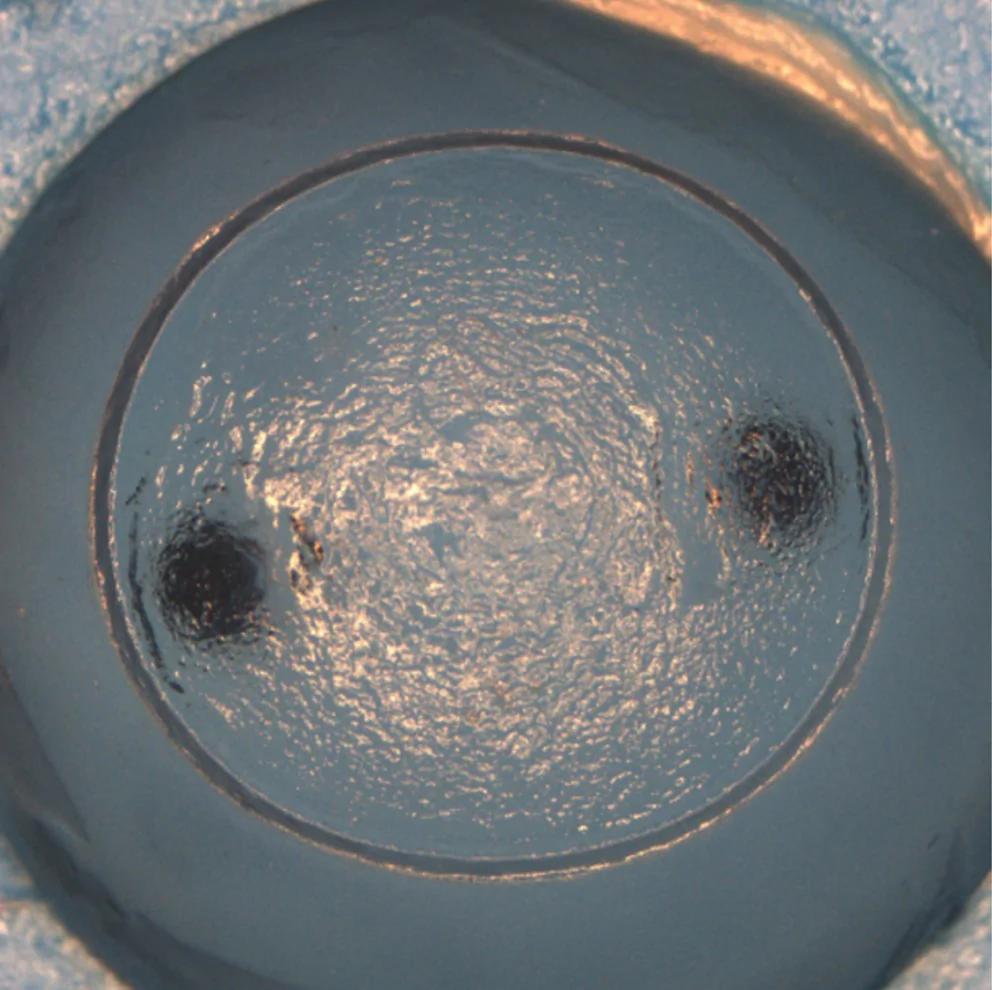
Stromal surface after extraction of the lamella cut with the frugal source. Parameters: 5 μJ/pulse, pulse duration 1.5 ps, spiral diameter 8 mm, spiral step size 2.5 μm, cutting depth 300 μm.

**TABLE 2:** Evaluation of the force required to lift the anterior lamellar (ease of extraction) depending on step size (in μm) and pulse duration (in ps). The colour code is used to indicate to the reader the qualitative value of the result obtained for the criterion in question. So, green means a good score, orange an average score, and red a bad score.

	Pulse duration (ps)	
	1.5	2.8
Step size (μm)		
2.5	3	3
5	2	2
10	1	1

**TABLE 3:** Evaluation of the cutting quality of an ALK depending on step size (in μm) and pulse duration (in ps). The colour code is used to indicate to the reader the qualitative value of the result obtained for the criterion in question. So, green means a good score, orange an average score, and red a bad score.

	Pulse duration (ps)	
	1.5	2.8
Step size (μm)		
2.5	8	7
5	7	6
10	2	2

The extraction of the anterior lamella after cut using the Helix Surgical source is shown in Video S1. Regarding the ease of lamella detachment, we observed no difference in effectiveness between the two pulse durations studied (1.5 and 2.8 ps) with a 7% *P/A ratio*. Only the step size influenced the ease of extracting the anterior lamella. A step size of 2.5 μm was identified as the ideal value.

### 2.4 Discussion

The primary aim of our pump‐probe experiments was to characterize the cavitation bubbles generated in water by laser pulses from the frugal source and to compare these with those produced by the industrial source. To the best of our knowledge, this study provides the most detailed exploration of cavitation dynamics with both high temporal and spatial resolution.

Our observations revealed that the main effect of varying parameters is on the height of the cavitation bubble, with minimal changes in its width. This can be explained by the way laser energy is deposited near the focal point, primarily along the vertical axis defined by the confocal parameter [19]. As laser intensity increases—whether through higher energy, smaller spot size, or shorter pulse duration—nonlinear effects become more pronounced, causing energy deposition further upstream along this axis. Reducing the volume of energy deposition is crucial, as it can minimize stromal collagen lamellae disruption, improve cutting precision, and reduce undesirable side effects of laser interaction.

Precision along the *Z*‐axis, enhanced through better confinement of the laser‐material interaction, is critical for improving spatial selectivity. This would allow for deep lamellar cuts while avoiding damage to the endothelium. Since the cutting plane is essentially formed by the assembly of cavitation bubbles, reducing their size is key to achieving more uniform and precise cuts. Stromal bed cutting is currently performed with pulse energies below 1 μJ while staying in the femtosecond regime, our results suggest that the picosecond regime offers additional benefits. Specifically, the frugal source generates smaller cavitation bubbles with higher energy density at the focal point.

Additionally, we observed that introducing a *P/A ratio* of around 20% further reduces the observed bubble size for a given energy, numerical aperture, pulse duration, and spot size. This parameter, which we initially thought to be detrimental due to imperfect temporal compression of the pulse, is intrinsically related to the structure of the frugal laser source and appears to give it an advantage over conventional sources.

The use of the frugal source for laser‐assisted anterior lamellar keratoplasties has demonstrated its ability to perform precise three‐dimensional cuts with defined parameters, including pulse durations in the picosecond range and pulse energies ranging from 5 to 6 μJ. This configuration not only produced uniform cuts but also facilitated lamella extraction. However, increasing the step size from 2.5 to 5 or 10 μm with a pulse duration of 1.5 ps resulted in an uneven distribution of cavitation bubbles, complicating lamella detachment and making extraction more difficult. Therefore, optimizing the cutting trajectory and pulse‐to‐pulse overlap is essential for achieving fine‐step incisions while maintaining an execution time similar to current commercial laser systems, which typically complete anterior lamellar keratoplasties in about 10 s (compared to 47 s with the frugal laser source).

When using a pulse duration of 2.8 ps, we found that cutting quality remained acceptable with a 2.5 μm step size, but a significant degradation occurred with larger step sizes, adversely affecting both cutting precision and ease of extraction. This narrower process window results from the lower peak intensity at 2.8 ps. These findings underscore the challenge of finding the optimal set of parameters in the picosecond regime and highlight the narrow window of effective conditions necessary to produce satisfactory results.

These results demonstrate that corneal cutting can be carried out with a cost‐effective all‐fiber picosecond laser source. However, the operating parameters need to be optimized to improve cut quality in order to compete with commercial systems that incorporate a femtosecond laser. Indeed, even if this work is not supported by some topography measurements of the stromal bed after laser treatment, the surface roughness of the stromal bed can be assessed with a measuring microscope within a range of 1–10 μm. These values are above the surface roughness ones reported in the scientific literature using a commercial femtosecond laser‐based system for LASIK operations [25, 26, 27], which are more within a range of 0.2–1 μm. However, one must keep in mind that each laser system for LASIK operations has its own optimized set of parameters in terms of optical setup, scanning device, scanning pattern, scanning parameters, and laser parameters, so a direct comparison of cutting quality is always an issue.

## 3 Conclusion

In this study, we investigated the potential of picosecond laser technology for corneal surgery, focusing on its ability to create precise cavitation bubbles and achieve superior cutting quality. Our research aimed to evaluate the characteristics of these bubbles and their impact on overall cutting performance compared to traditional femtosecond lasers.

Our pump‐probe experiments revealed that picosecond laser pulses produce smaller cavitation bubbles than those generated by femtosecond lasers. This is due to the enhanced concentration of excited electrons in the vicinity of the focal point with few picosecond pulses. Increased precision in bubble formation improves control over the cutting process, which is essential for achieving high‐quality corneal stromal cuts. This advancement indicates that picosecond lasers could surpass current technologies in terms of precision.

When examining cutting performance, the frugal picosecond source demonstrated that the optimal results are achieved with a pulse duration of 1.5 ps and a step size of 2.5 μm. This combination provided uniform cutting and facilitated lamella extraction. In contrast, increasing the step size to 5 or 10 μm, or extending the pulse duration to 2.8 ps, led to a decrease in cutting quality and more challenging extraction. These observations emphasize the importance of fine‐tuning laser parameters to maintain both precision and efficiency in corneal laser‐assisted surgeries.

By producing smaller cavitation bubbles and allowing fine cuts, picosecond lasers offer a promising alternative to the well‐established femtosecond lasers, with the potential to disrupt current practices in the field of laser‐assisted corneal surgery.

## 4 Experimental Section/Methods

### 4.1 Studied Sources

This study was carried out using two distinct laser sources operating at 1030 nm: the industrial Tangor laser and the frugal Helix Surgical laser, each coupled to a unique experimental setup.

The Tangor laser, developed by the French company Amplitude, is a high‐precision industrial ultrashort pulse laser operating at a wavelength of 1030 nm. It features advanced ultrafast amplifier technology, delivering femtosecond pulses with an energy per pulse of up to 500 μJ and peak powers reaching several gigawatts. This laser offers precise control over pulse duration, adjustable within a range of 0.5**–**5 ps, and supports a wide repetition rate range from 1 Hz to 2 MHz thanks to an external acousto‐optic modulator. It provides a maximum average power of 100 W, an excellent beam quality with an *M* ^2^ factor of 1.2, and an output beam diameter at 1/e^2^ of 3 mm. Additionally, it exhibits high temporal and spectral stability with energy fluctuations below 0.5% RMS.

The Helix Surgical laser is a frugal, portable system designed for laser‐assisted cataract surgery, representing a more cost‐effective alternative to traditional femtosecond lasers. Manufactured by the French company Helix Surgical, this system operates on battery power and integrates a fully fibered picosecond laser emitting at 1030 nm. The innovative frugal design reduces production costs by approximately 80% compared to conventional ophthalmic lasers. Key to this reduction is the factory‐fixed laser parameters, which eliminates the need for complex adjustments by the operator. Despite its lower cost, the Helix Surgical laser maintains comparable performance to traditional systems, with an average power of 3.6 W and a repetition rate of 320 kHz. However, it exhibits a slight degradation in its pulse temporal profile compared to high‐end lasers like the Tangor. The output beam is perfectly collimated and its beam diameter at 1/e^2^ is 2.5 mm. An external acousto‐optic modulator (MT80‐A1,5‐1064, AA Electronic) is used to trigger the laser and to reduce the effective repetition rate of the Helix Surgical source.

### 4.2 Experimental Setup for Pump‐Probe Shadowgraphy

To explore the dynamics of laser‐induced cavitation, we utilized a pump‐probe experimental setup. Each laser pulse, with a wavelength of *λ* = 1030 nm, was first divided into two beams using a beam splitter cube, creating the “pump” and “probe” pulses. Each followed distinct optical paths to the target. The energy distribution between the pump and probe, and individually on each optical path, was adjusted using half‐wave plates *λ*/2 and polarizing cubes. The pump pulse was directed through a specific optical path, including a beam expander and a microscope objective (M Plan APO NIR ×10/0.26, Mitutoyo) for focusing on the target (below the water surface). The beam arrived vertically on the target (Figure 6, red beam). The probe pulse was directed through a second optical path including a delay line and was then converted into a second harmonic (SHG) pulse with a wavelength of *λ* = 515 nm. This frequency conversion from near‐infrared to visible (green) light allowed for the illumination and observation of the interaction zone (Figure 6, green beam). The delay line is composed of two square retroreflecting hollow roof prism mirrors mounted on a mobile stage (Thorlabs). The position of this stage, with a travel range of 30 cm and a positioning accuracy of less than 10 μm, was controlled by specialized software (Kinesis, Thorlabs) from the control computer. This setup extended the probe's path by a distance of up to 2.40 m (four round‐trips), resulting in an additional propagation time Δ*t* (delay) of 8 ns.

**FIGURE 6:**
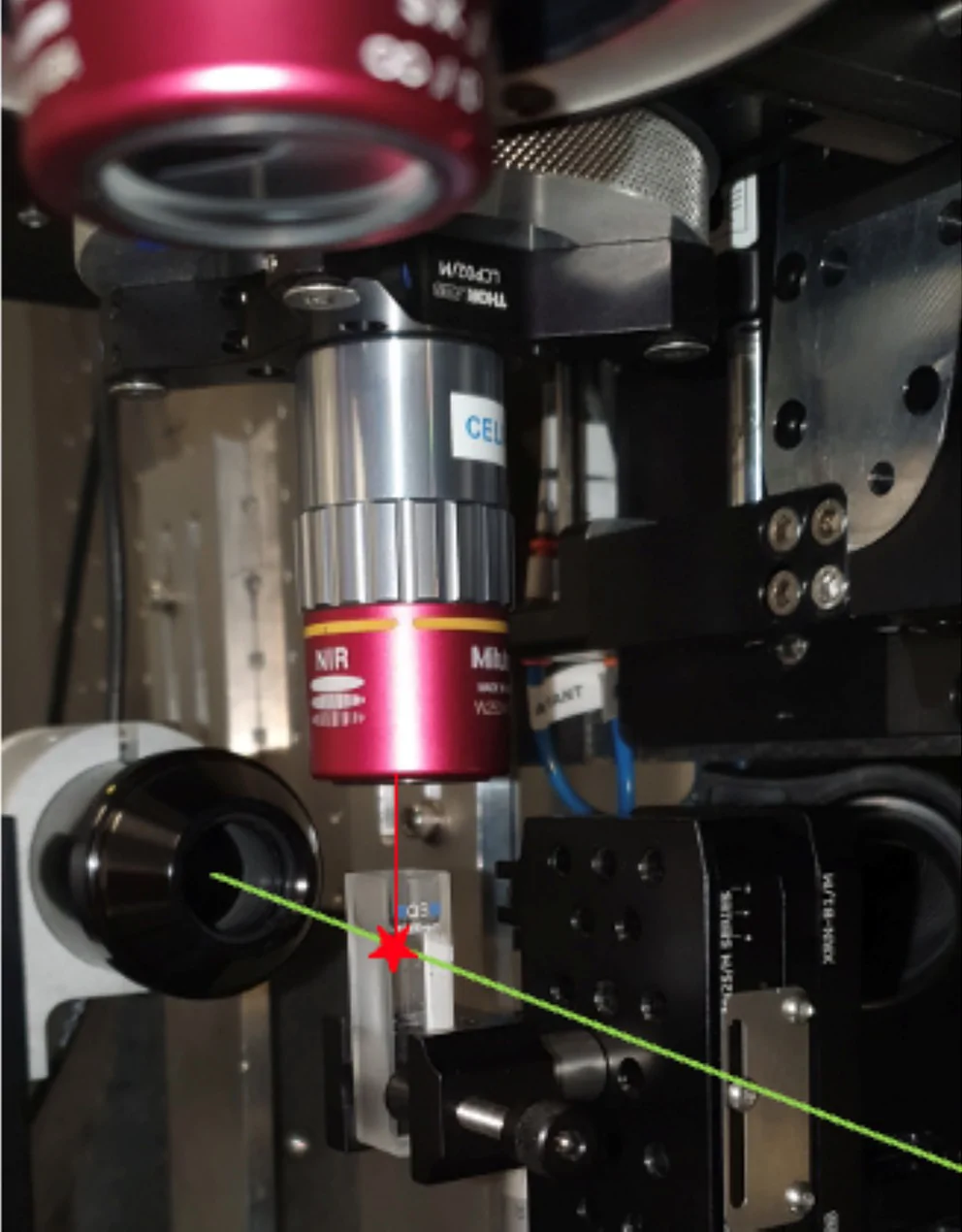
Experimental station, front view of the target: Quartz cell containing water (in the middle). The paths of the pump (red) and probe (green) intersect at the location of the generated cavitation bubble (star).

This experimental setup allowed for the study of laser‐material interactions (or, in this case, laser interactions with water) over a period of 8 ns. This configuration enabled us to reconstruct a detailed, frame‐by‐frame film of the first 8 ns of bubble formation, with a precision of 264 fs. The target used was a quartz cell containing water, with internal dimensions of 10 × 10 × 30 mm. This cell was mounted on the *XY* stages of the experimental station. Its precise positioning relative to the pump and probe was adjusted using the station's motorized *XY* stages and a manual micrometric *Z* stage.

The long‐distance microscope (model KX with MX‐6 objective, InfiniMax) used as a sideview imaging system was equipped with two bandpass filters at 515 nm, and a monochrome CMOS camera (acA1440‐220um, 1440 × 1080 pixels, 220 fps, Basler). The filters, placed before the camera, prevented the 1030 nm infrared radiation from the pump from reaching the sensor, thus avoiding saturation. The camera was controlled by Pylon software (Basler), which allowed for the configuration of acquisition parameters such as gain, delay and exposure time. The camera was triggered by the external laser modulator signal via a delay generator (Tombak, Aérodiode). Due to the speed difference between the laser light and the electrical signal, the first pulse of each series could not be captured. In practice, the laser pulse (*n*) triggered the recording of the image of the microbubble produced by the subsequent pulse (*n* + 1). The positioning of the imaging system was accomplished using a set of three independent manual micrometric stages (*XYZ*).

### 4.3 Image Processing and Bubble Size Measurement

The obtained images were processed using ImageJ software (National Institutes of Health) following the protocol summarized below:

1. Import of raw image in .tiff format,
2. Automatic binarization,
3. Skeletonization (automatic contour tracing) and selection of the area of interest,
4. Automatic measurement of the dimensions of the selected area (in pixels).

The measurement uncertainty was estimated at ±5 μm, encompassing both the repeatability of bubble size measurements in ImageJ and the reproducibility of results in unfavorable scenarios where the software fails to recognize the area of interest, requiring manual selection.

### 4.4 Calculation of the Theoretical Energy Required to Vaporize a Volume of Water Similar to an Oblong‐Like Bubble Volume

It is a well‐established fact that the energy required to vaporize 1 L of water ($E_{1\;\text{litre}}^{\mathrm{vap}}$) is about 2600 kJ starting from ambient temperature (20°C) and under ambient pressure (1 atm). From pump‐probe shadowgraphy images in Figure 1, we assume that the shape of the laser‐induced bubble can be considered as an oblong one (so, two half‐spheres plus a cylinder). Thus, the volume of the bubble can be estimated as follows:

(2) $$V_{\text{bubble}}=\frac{4}{3}\times \pi \times {\left(\frac{W_{b}}{2}\right)}^{3}+\pi \times {\left(\frac{W_{b}}{2}\right)}^{2}\times \left(H_{b}-W_{b}\right)$$

where $W_{b}$ and $H_{b}$ are the bubble width and height, respectively. And, the theoretical energy to vaporize such a water volume can be estimated as follows:

(3) $$E_{\text{bubble}}^{\mathrm{vap}}=E_{1\;\text{litre}}^{\mathrm{vap}}\times V_{\text{bubble}}$$

### 4.5 Studied Parameters

The main studied parameter was the pulse's temporal profile. This variable can be detailed into two sub‐entities: pulse duration (defined as the full width at half maximum of the intensity curve as a function of time assuming a Gaussian pulse) and the quality of temporal pulse compression (i.e., the temporal contrast of the pulse). While it is possible to set the pulse duration of the frugal laser system, this affects the quality of its compression, unlike the industrial Tangor laser system in which the pulse duration can be adjusted without degrading compression. To describe compression quality, we defined a *P/A ratio*, with *P* being the normalized area of the pedestals (Figure 7, the green hatched area) and *A* being the normalized area under the measured intensity curve (Figure 7, full area below the blue curve) as a function of time. The closer this ratio is to zero, the better the pulse compression. Conversely, a higher ratio indicates a poorer temporal profile quality. Secondary parameters included energy per pulse and depth below the water surface.

**FIGURE 7:**
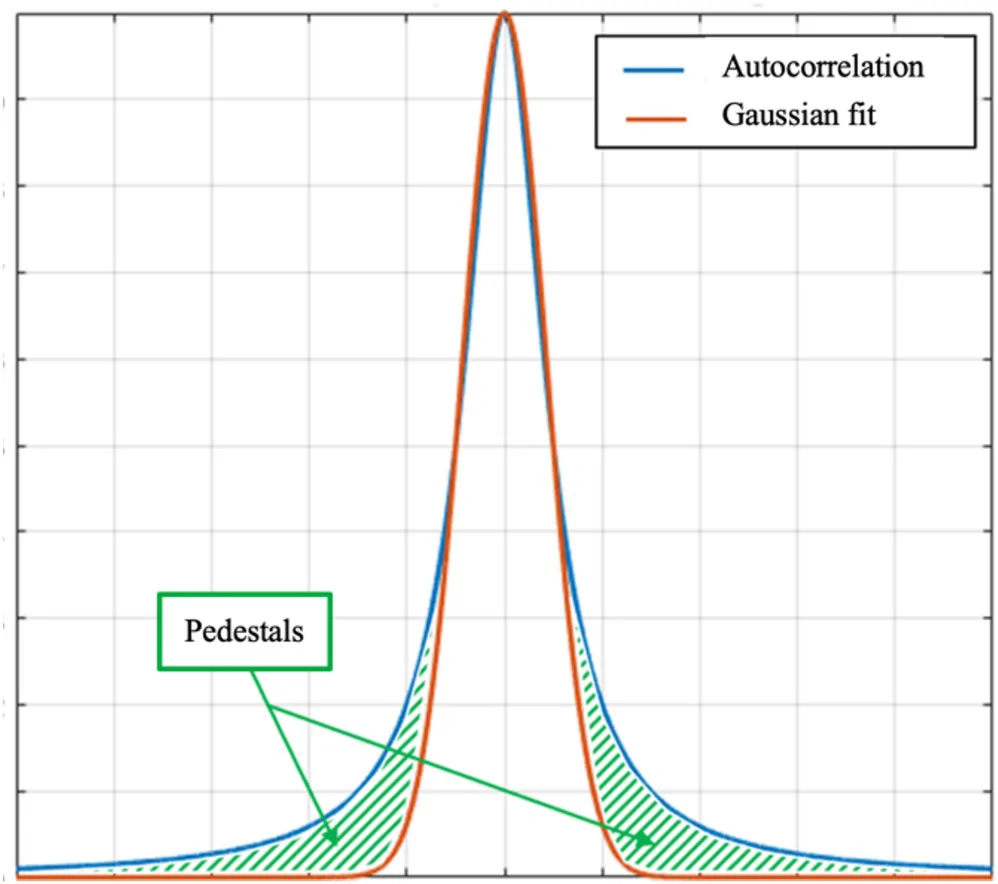
Diagram of the temporal pulse profile. The blue curve represents the measured pulse intensity over time. The red curve represents the Gaussian fit to the measured intensity. The pedestals represent the difference in area under the curve between the autocorrelation trace (blue) and the Gaussian fit (red).

The studied parameters are summarized in Table 4. Prior to each acquisition, these parameters were verified using metrology instruments: an autocorrelator (ROC‐PS3‐1030, range 300 fs—3 ps, FemtoEasy) to measure pulse duration and characterize compression, and a powermeter (UP19K‐50F‐W5 thermopile power sensor with Maestro controller, Gentec) to assess the energy per pulse.

**TABLE 4:** Summary of the studied parameters and the considered values.

	Variable (unit)	Considered values
Main parameter: Temporal profile	Pulse duration (ps)	Helix surgical: 0.84–0.88–1.20–2.06 Tangor: 0.48–0.85–1.00–1.20–1.51–2.00–2.51–3.01–3.50–4.00–4.50–5.01
	P/A ratio (%)	Helix surgical: 11–18–19–22 Tangor: 3
Secondary parameters	Depth below surface^a^ (μm)	100–200–300–400–500–600–700–800–900–1000
	Energy per pulse (μJ)	Helix surgical: 0.9–1.0–1.2–1.5–2.0 Tangor: 0.5–0.7–0.75–0.9–1.0–1.1–1.2–1.5–1.7–1.8–2.0–2.2–2.5–3.0

^a^ The refractive index of water (*n*_water_ = 1.33) was taken into account for depth calculation.

### 4.6 Default Parameters

Unless otherwise noted, the bubbles described and studied in the manuscript are formed by focusing the laser beam with the microscope objective with an effective numerical aperture (NA) of 0.22, resulting in a 6.0 ± 0.1 μm spot size, measured at 1/e^2^ in air and at the focal point using a beam analyzer (DAT WinCamD‐XHR, pixel size 5.5 μm, Dataray) along with a ×10 calibrated home‐made magnification system. Bubbles are observed 4.0 ns after cavitation bubble formation and 300 μm below the water surface (optical depth).

### 4.7 Statistical Analysis

The statistical analysis of the results was carried out in several stages using multiple consecutive tests. Every experiment was produced 10 times per set of parameters (pulse duration, energy per pulse). Statistical significance was assessed using a significance level of *α* = 0.05, meaning that results with *p*‐values below this threshold were considered statistically significant. The *p*‐value is the probability of obtaining results as extreme as, or more extreme than, the observed results, assuming that the null assumption is true. The alpha risk corresponds to the probability of a Type I error, that is, incorrectly rejecting the null assumption.

Sequential tests applied:

- Normality Test (Shapiro–Wilk [28], *α* = 0.05): We test whether each series follows a normal distribution. Normality is rejected if *p* < 0.05.
- Test for Equality of Variances (Levene [29], *α* = 0.05): If both series are normally distributed, we test whether their variances are equal. Equality of variances is rejected if *p* < 0.05.
  - Student's *t*‐test [30] (if distribution is considered normal). Depending on the result of the Levene test, we choose the *t*‐test with or without the assumption of equal variances.
  - Mann–Whitney nonparametric test [31] (if distribution is considered nonnormal).

### 4.8 Corneal Ablation Quality

Secondly, we were able to perform some cuts using the frugal source. The corneal cutting experiments were carried out using a galvo scanner (LS‐Scan XY 20, Lasea) equipped with a telecentric lens (focal length 30 mm, entrance pupil 14 mm, max. numerical aperture 0.22). The spot size at 1/e^2^ is also 6.0 ± 0.1 μm. The pig cornea is mounted on an artificial anterior chamber and cut through a 1 mm‐thick aplanation glass (sodalime). The chosen trajectory is that of anterior lamellar keratoplasty. This trajectory is composed by a horizontal spiral and a vertical helicoidal pattern. It allows us to test the frugal Helix Surgical source's ability to cut in three dimensions: along the *X* and *Y* axes for spiral producing the deep plane, and along the *X*, *Y*, and *Z* axes for the helicoidal rising towards the epithelial surface, cutting the edges of the lamella. Additionally, this trajectory includes another spiral that provides a flat stromal surface, allowing easier analysis under an optical microscope. Each turn of the spiral is composed of 40 contiguous segments. The scanning velocity is set to 180 mm/s for the spiral below a diameter of 2 mm and for the helicoidal trajectory, and to 640 mm/s for the spiral above 2 mm in diameter. The scanning velocity is limited by the dynamic of the galvo scanner. The step size (pitch) of the spiral can be 2.5, 5, or 10 μm. The repetition rate of the Helix Surgical source is set to 320 kHz, and the pulse energy on target to 5 μJ.

In the minute following the laser cutting of an 8 mm diameter and 300 μm thickness lamella in the anterior corneal stroma, a micromanipulator was introduced into the interface between the stromal bed and the lamella to be extracted. The force required to lift the lamella and detach its edges around the entire perimeter was quantified using a dedicated score (Table 5). If the manipulator was unable to detach the lamella, a Troutman forceps was then used. The resistance perceived by the surgeon during this maneuver is directly related to the consistency and continuity of the cavitation bubbles formed in the cornea, indicating the effectiveness and uniformity of the laser cut.

**TABLE 5:** Cut effectiveness score, based on the instrument used to extract the lamella.

Instrument used	Score
Spatula (no resistance)	3
Spatula (with resistance)	2
Troutman forceps	1
Total	out of 3

Furthermore, the cutting quality was quantified using a dedicated score (Table 6). The stromal bed and the lamella edges were carefully examined using a binocular loupe (model SMZ745T, Nikon) and an optical microscope (MF‐B1010D, Mitutoyo) equipped with a ×20 objective (ML ×20, NA 0.42, WD 20 mm, Mitutoyo). For each cut, 50 areas of the stromal bed were observed under the microscope, each covering a field of 420 × 350 μm. The distribution of the areas of interest was as follows: 10 in the central 2 mm and 10 in each of the four peripheral quadrants.

**TABLE 6:** Cut quality score.

Criterion		Aspect	Score
A	Stromal regularity^a^	Regular	1
		Irregular	0
B	Edge regularity^a^	Regular	1
		Irregular	0
C	Microscopic stromal regularity^b^	Excellent	2
		Good	1
		Poor	0
D	Proportion of irregular zones^b^, ^c^	< 10%	2
		10%–25%	1
		> 25%	0
E	Irregularity localization^b^	None	2
		Peripheral	1
		Central/diffuse	0
Total			out of 8

^a^ Binocular loupe observation, ×5 magnification.

^b^ Optical microscope examination, ×20 magnification.

^c^ Among 50 observed zones, 10 in the central 2 mm, and 10 in each peripherical ¼ quadrant.

## Funding

This research was supported by the Banque Française des Yeux and the Fondation de France.

## Conflicts of Interest

Florent Deloison is a co‐founder, shareholder, and current Chief Executive Officer of Helix Surgical company, that provided the picosecond laser used in this investigation. David Touboul serves as a member of the scientific advisory board of Helix Surgical. The remaining authors declare that the research was conducted in the absence of any commercial or financial relationships that could be construed as a potential conflict of interest.

## Supporting information


**Video S1:** Extraction of an anterior lamella after cut using the Helix Surgical source.

## Data Availability

The data that support the findings of this study are available from the corresponding author upon reasonable request.
